# Serum and urine ANGPTL8 expression levels are associated with hyperlipidemia and proteinuria in primary nephrotic syndrome

**DOI:** 10.1186/s12882-021-02350-w

**Published:** 2021-04-14

**Authors:** Yue Li, Qingju Liu, Chengdong Kang, Weijing Cui, Zichuan Xu, Fu Zhong, Xia Gao

**Affiliations:** 1 Graduate School, Gansu University of Chinese Medicine, Lanzhou city, 730000 China; 2 Departmentof Pediatrics, the Fifth People’s Hospital of Chengdu, Chengdu city, 611130 China; 3 Department of Nephrology, Guangzhou Women and Children’s Medical Center, Guangzhou city, 510623 China; 4 Pediatric Department, Gansu Provincial Hospital, Lanzhou city, 730000 China

## Abstract

**Background:**

This study aimed to investigate the expression characteristics of ANGPTL8 in patients with primary nephrotic syndrome and its possible correlation with hyperlipidemia and proteinuria.

**Methods:**

ANGPTL8 levels were determined using an enzyme-linked immunosorbent assay in 133 subjects with PNS and 60 healthy controls.

**Results:**

Compared with healthy controls, subjects with primary nephrotic syndrome had higher levels of serum and urine ANGPTL8 (*P* < 0.001). In primary nephrotic syndrome patients, serum ANGPTL8 was positively correlated with cholesterol (r = 0.209, *P* < 0.05) and triglycerides (r = 0.412, *P* < 0.001), while there was no correlation with 24 hUTP. Urine ANGPTL8 was positively correlated with high-density lipoprotein cholesterol (r = 0.181, *P* < 0.05) and was significantly negatively correlated with creatinine (r = − 0.323, *P* < 0.001), eGFR (r = − 0, *P* < 0.001) and 24 hUTP (r = − 0.268, *P* = 0.002). Interestingly, the urine ANGPTL8 concentrations in membranous nephropathy and mesangial proliferative glomerulonephritis pathological types were different.

**Conclusions:**

Serum and urine ANGPTL8 levels in primary nephrotic syndrome patients were correlated with blood lipid levels and proteinuria, respectively, suggesting that ANGPTL8 may play a role in the development of primary nephrotic syndrome hyperlipidemia and proteinuria.

## Background

The typical clinical manifestations of PNS include massive proteinuria, hypoproteinemia, edema and/or hyperlipidemia [1]. Of these, marked proteinuria is the core clinical manifestation, and its severity often parallels the degree of hyperlipidemia [2–4]. The mechanism of PNS complicated with hyperlipidemia is not well understood.

Members of the angiopoietin-like protein (ANGPTL) family are mainly involved in vascular endothelial genesis and the regulation of lipid metabolism [5, 6]. Recently, ANGPTL3 and ANGPTL4 have been shown to be involved in podocyte injury [7–9]. Our previous studies demonstrated that high expression levels of ANGPTL3 in glomerular podocytes leads to rearrangement of the podocyte cytoskeleton and increases podocyte activity, which induces proteinuria [10, 11]. In addition, serum ANGPTL3 levels were found to be significantly positively correlated with triglyceride and cholesterol levels in patients with nephrotic syndrome [12].

We also found that ANGPTL4 was positively correlated with TG levels in patients with hyperlipidemia-related kidney injury but negatively correlated with the level of proteinuria in patients [13]. An increasing number of studies have also revealed that ANGPTL4 participates in the occurrence and development of PNS [14]. ANGPTL4 expression in the kidneys of FSGS mice induced by adriamycin was markedly upregulated [15].

As a newly recognized ANGPTL family member, ANGPTL8 has been found to be highly expressed in mouse liver and brown adipose tissue [16]. Recent studies have detected ANGPTL8 in human and mouse serum [17], and moderate levels have been found in human urine [18]. Studies have confirmed that ANGPTL8 can promote the molecular cleavage of ANGPTL3, and it binds to the N-terminus of ANGPTL3 to form an N-terminal complex, which synergistically inhibits the activity of LPL and increases TG levels [19]. Moreover, recent studies have confirmed that ANGPTL8, ANGPTL4, and ANGPTL3 synergistically interact to participate in fatty acid transfer [20].

Based on the above findings, here, we investigated the characteristics of serum and urine ANGPTL8 levels in PNS patients and further examined their relationship with lipid metabolism parameters and the severity of urinary protein.

## Methods

### Subjects

This cross-sectional study was carried out between November 2017 and November 2019 in Gansu Provincial People’s Hospital. The study was approved by the Medical Ethics Committee of Gansu University of Traditional Chinese Medicine (No. syll20160037), and all methods were carried out in accordance with relevant guidelines and regulations. All participants provided oral and written informed consent prior to participation. A total of 193 subjects were recruited, including 133 patients with PNS and 60 healthy controls (HCs). Subjects underwent a detailed medical history record review, medical examination, comprehensive urine analysis, routine blood testing, endocrine profiling, biochemical analysis and ultrasound examination of the urinary system. We performed autoantibody tests, an HBV assay, and immune globulins assay, to identify secondary nephropathy. Renal biopsy was carried out in 78 patients based on their disease characteristics.

The PNS patient inclusion criteria were as follows: heavy proteinuria [24-h urine total protein (24 hUTP) > 3.5 g or urine protein/creatinine ratio > 3.0 mg/mg or 24 hUTP > 50 mg/kg] and hypoalbuminemia [serum albumin (ALB) ≦25 g/L], and various degrees of edema and hyperlipidemia [21].

The HCs had no concomitant health problems, and fasting blood lipid levels [22] [cholesterol (CHOL) < 200 mg/dl, triglycerides (TG) < 150 mg/dl, and low-density lipoprotein cholesterol (LDL-C) < 130 mg/dl] and urinary proteins (urinary microalbumin ≤150 mg/dl or negative urine qualitative test) were within the normal range.

The exclusion criteria were as follows: PNS patients without the required clinical and laboratory data, those with secondary nephrotic syndrome, previous history of other acute or chronic kidney disease, patients with abnormal ultrasound examination of the urinary system (e.g., deformities, cysts, hydrops, stones), identified acute or chronic illness (diabetes mellitus, thyroid dysfunction, polycystic ovary syndrome, obesity, fatty liver, familial hypercholesterolemia), and other systemic diseases, such as hematological diseases, cardiovascular diseases, connective tissue diseases, tumors, and obvious infections.

### Anthropometric and laboratory measurements

The medical records of the following parameters were collected for the study: age, sex, body mass index (BMI), calculated as weight divided by height squared (kg/m2), blood pressure, disease state including initial treatment, pathology results of renal biopsy provided by Guangzhou KingMed Diagnostics (an independent ISO15189-certified clinical laboratory) including minimal change disease (MCD), focal segmental glomerulosclerosis (FSGS), membranous nephropathy (MN), and mesangial proliferative glomerulonephritis (MsPGN). Also collected were the levels of serum CHOL, TG, high-density lipoprotein cholesterol (HDL-C), LDL-C, ALB, creatinine (CREA), urea (Ur) [measured by automated biochemical analyzer (ARCHITECT c1600, Abbott, USA)], 24 hUTP, urine creatinine (UCr) [measured by automated chemiluminescence immunoanalyzer (UniCel DxI 800, Beckman-Coulter, USA)], and urine protein [measured by automated urine analyzer (FUS-2000, DIRUI, China)]. All assays were performed according to routine procedures specified by the clinical laboratory center (ISO15189 certified) of Gansu Provincial People’s Hospital. The severity of proteinuria was divided into three categories according to the 24 hUTP level: mild (≤ 1 g/d), moderate (1 ~ 3.5 g/d), and severe (≥ 3.5 g/d) proteinuria. The estimated glomerular filtration rate (eGFR) was calculated from the serum creatinine level using the Modification of Diet in Renal Disease (MDRD) study equation [23].

### Enzyme-linked immunosorbent assay (ELISA)

Blood samples were collected from the antecubital vein of subjects between 07:30 and 09:00 a.m. after an overnight fast (at least 10 h). The collected blood was coagulated for 30 min at 4 °C and then centrifuged for 20 min at 3000 rpm, 4 °C. A clean, midstream first-morning urine sample was also obtained from all subjects. The assay samples were immediately stored at − 20 °C prior to ELISA, and repeated freeze–thaw cycles were avoided. The serum and urine ANGPTL8 concentrations were measured with ELISA kits (Jiangsu Meimian Industrial Co., Ltd., Jiangsu, China, Catalog No. E1766H1), and the procedures were conducted in compliance with the manufacturer’s protocol.

### Statistical analysis

The distribution pattern of continuous variables was tested for normality using the Kolmogorov–Smirnov (*n* ≥ 100) or Shapiro–Wilk test (*n* < 100). For normally distributed variables, the data are presented as the mean ± standard deviation (S.D.). Differences were assessed by independent samples t-test, two-tailed Student’s t-test, or one-way ANOVA. Pearson correlation coefficients were estimated to determine the correlation. For non-normally distributed variables, the data are presented as the median [interquartile range]. Differences were assessed by the Mann-Whitney U test, the Kruskal-Wallis test, or Kruskal-Wallis one-way ANOVA, and correlations were analyzed by the Spearman correlation method. Categorical variables were reported as frequencies or percentages. Differences were assessed by the Pearson χ2 test or the continuity modified χ2 test. For grade variables, differences were assessed by the Wilcoxon signed-rank test. A two-sided *P* value < 0.05 was considered statistically significant. All statistical analyses were carried out with SPSS software (version 26.0).

## Result

### Baseline characteristics of the patients

Table 1 summarizes the clinical characteristics of the included patients. A total of 133 patients were enrolled in the PNS group; 94 males (70.68%) and 39 females (29.32%), and the male to female ratio was 2.41. Overall, 33 patients were less than 18 years of age, while there were 100 adult PNS patients. Regarding disease status, there were 48 initial cases, 51 partial remission cases and 34 relapse cases. Sixty subjects were enrolled in the control group; 40 males (66.67%) and 20 females (33.33%), with a male to female ratio of 2.41. The PNS patients had typical manifestations, including massive proteinuria [24 h proteinuria of 5.989 (2.297, 9.140)], hypoalbuminemia, and hyperlipidemia (Table 1). There were no differences in sex, age or blood pressure between the patient group and the HC group (*P* > 0.05).

**Table 1:** Anthropometric and laboratory characteristics of healthy individuals and patients with PNS

Variables	PNS *n* = 133	Controls *n* = 60	*P* value
Sex (F/M)	94 / 39	40 / 20	0.576
Age (years)	33.23 ± 20.16	36.05 ± 16.40	0.276
BMI (kg/m2)	22.94 ± 2.78	22.24 ± 1.75	0.051
SBP (mmHg)	120.00 (107.50, 129.00)	117.00 (108.25, 126.75)	0.466
DBP (mmHg)	73.39 ± 10.03	73.50 ± 8.32	0.941
CHOL (mmol/L)	8.25 (6.42, 10.33)	4.39 (3.74, 4.95)	< 0.001*
TG (mmol/L)	2.77 (1.91, 3.29)	1.11 (0.96, 1.43)	< 0.001*
HDL-C (mmol/L)	1.66 (1.25, 1.98)	1.31 (1.19, 1.48)	< 0.001*
LDL-C (mmol/L)	5.11 (3.61, 6.64)	2.51 (2.13, 2.91)	< 0.001*
ALB (g/L)	23.40 (17.90, 28.95)	46.25 (44.13, 47.96)	< 0.001*
CREA (μmol/L)	62.50 (46.60, 78.10)	58.65 (50.13, 76.15)	0.891
Ur (mmol/L)	6.10 (4.50, 8.30)	4.26 (3.67, 5.39)	< 0.001*
Urine protein	1 ~ 4	ngegative	< 0.001*
24hUTP (g/d)	5.989 (2.297, 9.140)	–	
Serum ANGPTL8 (ng/ml)a	28.29 (22.31, 31.30)	23.21 (19.55, 25.11)	< 0.001*
Urine ANGPTL8 (ng/ml)a	42.36 (37.45, 76.44)	36.38 (33.59, 39.41)	< 0.001*

F: female, M: male. In the qualitative test of urine protein, ‘0’ represents ‘-’, ‘1’ represents ‘+’, ‘2’ represents ‘++’, ‘3’ represents ‘+++’, ‘4’ represents ‘++++’

* *P* < 0.05 was considered significant

### Serum and urine ANGPTL8 levels were significantly increased in PNS patients

**Fig. 1:**
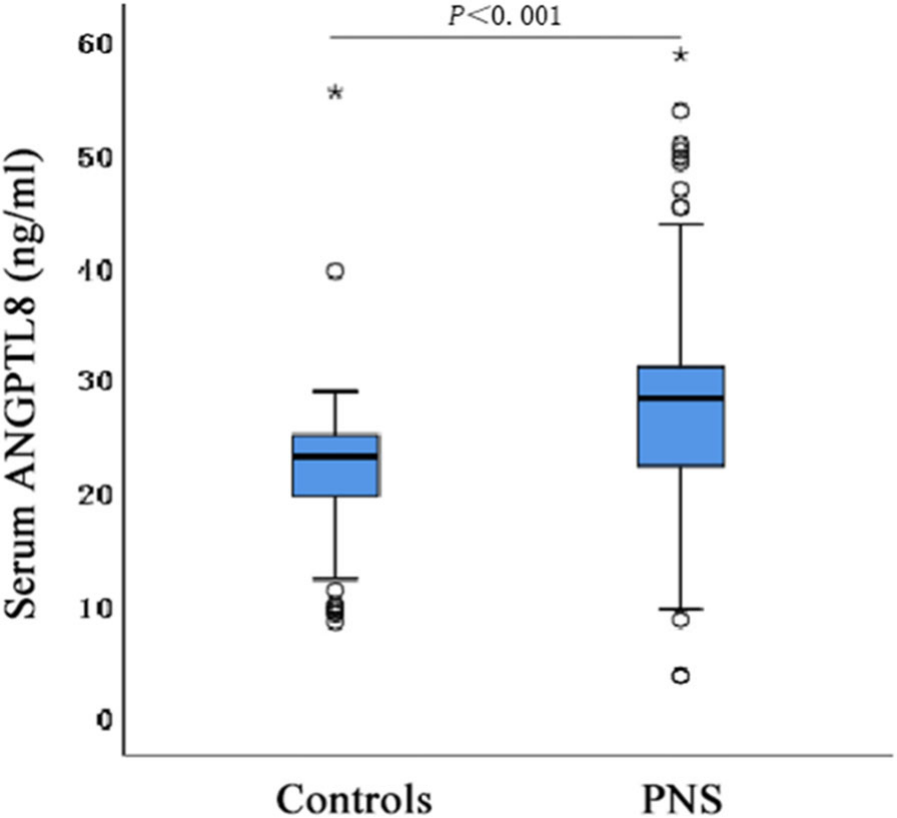
Comparison of serum ANGPTL8 levels box-plot between PNS group and control group, patients with PNS had significantly higher serum ANGPTL8 levels than healthy controls (*P* < 0.001)

**Fig. 2:**
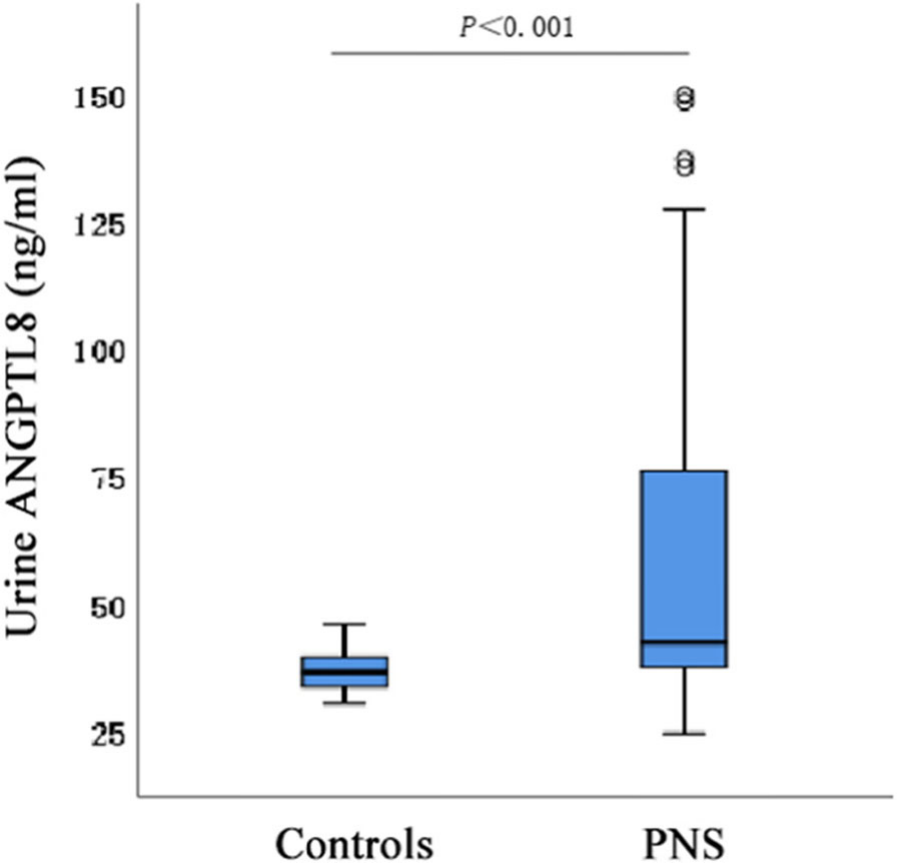
Comparison of urine ANGPTL8 levels box-plot between PNS group and control group, patients with PNS had significantly higher urine ANGPTL8 levels than healthy controls (*P* < 0.001)

Figures 1 and 2 show that PNS patients had significantly higher serum and urine ANGPTL8/UCr concentrations than the control group. However, no significant difference was found in the expression of serum ANGPTL8 and urine ANGPTL8/UCr in PNS patients with or without glucocorticoid treatment (Table 2).

**Table 2:** Comparison of ANGPTL8 levels of PNS patients by treatment condition

Treatment condition	Serum ANGPTL8 (ng/ml)	Urine ANGPTL8/UCr (ug/g)
Untreated(*n* = 82)	28.44 (22.71, 30.84)	56.31 (37.53, 101.51)
Treated (*n* = 51)	26.70 (21.53, 32.52)	60.18 (44.20, 107.56)
*P* value	0.926	0.345

### Urine ANGPTL8/UCr levels in PNS patients significantly changed with different degrees of proteinuria

The PNS patients were further grouped into three proteinuria groups according to the 24 hUTP level. As shown in Table 3, serum ANGPTL8 levels were not significantly different among the three proteinuria groups (*P* > 0.05), while urine ANGPTL8/UCr levels showed significant differences among the three proteinuria groups (*P* < 0.05).

**Table 3:** Comparison of ANGPTL8 levels in PNS patients by varying degrees of proteinuria

Degree of proteinuria	Serum ANGPTL8 (ng/ml)	Urine ANGPTL8/UCr (ug/g)
Mild (n = 8)	23.53 (14.97, 29.29)	74.17 (34.63, 178.57)
Moderate (*n* = 40)	28.19 (22.43, 31.07)	89.72 (45.28, 133.80) *
Severe (*n* = 85)	28.40 (22.34, 32.22)	50.47 (38.54, 80.20)
*P* value	0.283	0.019

* The significance values were adjusted by Bonferroni correction, which was statistically significant compared with the severe proteinuria (*P* < 0.05)

### Serum ANGPTL8 levels were correlated with blood lipid levels in PNS

As shown in Table 4, in PNS patients, the serum ANGPTL8 levels were significantly positively associated with CHOL (r = 0.209, *P* < 0.05) and TG levels (r = 0.412, *P* < 0.001), while no significant association was found between serum ANGPTL8 levels and 24-h UTP.

**Table 4:** Correlation between ANGPTL8 levels and main clinical indicators in patients with PNS

Variables	Serum ANGPTL8		Urine ANGPTL8/UCr	
	r	*p* value	r	*p* value
CHOL	0.209	0.016*	0.073	0.406
TG	0.412	0.000*	0.036	0.682
HDL-C	0.065	0.445	0.201	0.020*
LDL-C	0.129	0.140	0.113	0.193
ALB	−0.027	0.757	−0.068	0.434
CREA	−0.017	0.845	− 0.323	0.000*
Ur	0.115	0.188	−0.089	0.309
eGFR	−0.137 (− 0.119)	0.116 (0.813)	0.016 (0.352)	0.853 (0.000*)
24hUTP	0.087	0.321	−0.268	0.002*
BMI	−0.069	0.461	−0.206	0.028*

Spearman’s correlation analysis was used

r: Spearman’s correlation coefficient

* *P* value < 0.05 was significant correlation

The correlation analysis of urine ANGPTL8 and lipid metabolism indicators showed that urine ANGPTL8/UCr levels were positively correlated with HDL-C (r = 0.201, *P* < 0.05) and negatively correlated with CREA (r = − 0.323, *P* < 0.001), eGFR (r = − 0.352, *P* = 0.000) and 24 h UTP (r = − 0.268, *P* < 0.05) (Table 4).

### Urine ANGPTL8 levels were associated with PNS pathological type

We investigated the serum and urine ANGPTL8 levels in 78 PNS patients with renal biopsy pathology results, which included 21 MCD, 8 MsPGN, 38 MN and 11 FSGS patients. No significant differences in serum ANGPTL8 levels were found across the different PNS types. However, the urine ANGPTL8/UCr level was significantly different between the MN and MsPGN groups (*P* < 0.05, Table 5).

**Table 5:** Comparison of ANGPTL8 levels of PNS patients by different pathological types

Pathological types	Serum ANGPTL8 (ng/ml)	Urine ANGPTL8/UCr (ug/g)
MCD (*n* = 21)	25.76 ± 9.09	52.78 (44.21, 86.77)
MsPGN (n = 8)	26.42 ± 5.72	91.68 (53.16, 130.32) *
MN (*n* = 38)	26.00 ± 9.47	45.08 (31.06, 69.86)
FSGS (*n* = 11)	27.13 ± 6.79	76.01 (35.64, 134.98)
*P* value	0.977	0.010

* The significance values were adjusted by Bonferroni correction, which was statistically significant compared with the MN group (*P* < 0.05)

## Discussion

The molecular mechanism of massive proteinuria complicated with hyperlipidemia in PNS has not yet been elucidated. Recent studies have shown that the degree of hyperlipidemia in PNS is often parallel to the severity of proteinuria [24]. Hyperlipidemia symptoms occur in PNS patients and involve two main mechanisms. First, increased glomerular permeability results in massive urinary protein loss; as a result, this severe hypoproteinemia stimulates protein synthesis in the liver, leading to the increased production of lipoproteins. Second, a reduced concentration and decreased activity of lipid lipase (LPL) (a key rate-limiting enzyme in lipid catabolism) in the blood impairs the clearance of low-density lipoprotein (LDL) and very low-density lipoprotein (VLDL).

As the key inhibitors of LPL, the ANGPTL family members ANGPTL3 and ANGPTL4 interfere with LPL activity in different ways, leading to increased levels of TG, TC, and LDL and the development of hyperlipidemia [6]. Interestingly, these two molecules are also involved in the occurrence of PNS proteinuria [7, 10]. ANGPTL3 activates the podocyte integrin b3 receptor, leading to the activation of FAK/PI3K/ANTN4, and is involved in the mechanism of podocyte injury [9, 11]. Sialylated ANGPTL4, synthesized by podocytes, has been found to be closely related to podocyte damage [25]. Additionally, the degree of nephrotic proteinuria in ANGPTL4-knockout mice is significantly alleviated [25]. The increase in circulating ANGPTL4 promoted hypertriglyceridemia and decreased proteinuria in NS rats [25]. ANGPTL8 is a new member of the ANGPTL family that coordinately regulates the expression levels of ANGPTL3 and ANGPTL4. In this study, we investigated the features of ANGPTL8 expression in the serum/urine of PNS patients and its correlation with the lipid index and the severity of proteinuria.

ANGPTL8, a potent inhibitor of LPL activity, reduces the clearance of TG, thereby increasing the TG serum level [16]. A study on gene-edited mice showed higher levels of serum TG in ANGPTL8-overexpressing mice and lower levels in ANGPTL8-knockout mice [16, 17]. In the current study, we used ELISA to detect ANGPTL8 in the blood and urine of PNS patients. Compared with healthy controls, the PNS patients had significantly higher levels of serum and urine ANGPTL8, which implies a potent role of ANGPTL8 in PNS pathophysiology. Our data also suggest that ANGPTL8 functions in PNS hyperlipidemia. A further comparison of ANGPTL8 expression in initial and relapsed patients revealed no significant difference in serum and urine ANGPTL8 levels in the different disease states. However, this study did not investigate ANGPTL8 levels in PNS patients at different stages of drug treatment. Thus, future studies should clarify the influence of glucocorticoids on ANGPTL8 expression.

The correlation analysis showed that serum ANGPTL8 levels were positively associated with both CHOL and TGs, but not with HDL-C and LDL-C. These findings are inconsistent with reported results regarding ANGPTL8 levels in other kidney diseases. In patients with type 2 diabetes, serum ANGPTL8 was positively correlated with TGs and negatively correlated with CHOL and HDL. This finding indicates that diabetic nephropathy and PNS have distinctive pathogeneses.

A high BMI is a known risk factor for hyperlipidemia, and patients with long-term hyperlipidemia often have an elevated BMI. In previous studies of diabetic nephropathy, serum ANGPTL8 was found to be positively correlated with patient BMI [26]. The current study, however, excluded PNS patients with diabetes and found that the BMIs of the PNS and control groups were not significantly different. The R value of the correlation between ANGPTL8 and BMI was − 0.069, with a *P* value of 0.461. Why is ANGPTL8 not associated with BMI in PNS patients? Abnormal levels of blood lipids in PNS patients are always accompanied by kidney damage and can be alleviated with the improvement of proteinuria. We found that the BMI of PNS patients was not significantly different from that of healthy people. Therefore, no correlation between BMI and ANGPTL8 was found in PNS patients.

In the type 2 diabetes study [27], the ANGPTL8 level was found to be related to urinary albumin excretion, and there was a positive correlation between serum ANGPTL8 and urinary albumin/creatinine levels (r = 0.427, *P* < 0.001). In our study of PNS patients, there was no statistically significant difference between serum ANGPTL8 and 24 hUTP. Further analysis by PNS pathology type showed no significant changes in serum ANGPTL8. The pathogeneses of renal injury in PNS and diabetic nephropathy might be different, in particular, the role of metabolic factors in the two diseases. This might explain the different patterns of the ANGPTL8-proteinuria relationship in these two diseases.

Our study also detected ANGPTL8 in urine samples and found that ANGPTL8/UCr was positively correlated with the HDL level. ANGPTL8 is a small secreted protein that can pass through the glomerular filtration barrier and enter the urine. Therefore, urine ANGPTL8 reflects the amount of ANGPTL8 synthesized by the kidney and is related to the level of circulating ANGPTL8. However, additional investigation into the correlation between urine ANGPTL8 and HDL are needed. Because ANGPTL8 can be synthesized in the kidney, it will be necessary to analyze the relationship between urine ANGPTL8 and kidney injury indicators as a basic step in further mechanistic studies. We found that urine ANGPTL8/UCr was positively correlated with eGRF and negatively correlated with the degree of proteinuria, whereas no relationship was observed for serum ANGPTL8. Our data also showed that increases urine ANGPTL8 was associated with indicators of kidney injury. Further animal model studies are necessary to explore the interaction between synthesized ANGPTL8, ANGPTL3 and ANGPTL4 in the occurrence of podocyte injury.

The various renal pathological types exhibit different serum and urine ANGPTL8 levels. Urine ANGPTL8 levels were significantly higher in the MsPGN group than in the MN group; however, no difference was found for serum ANGPTL8, suggesting that urine ANGPTL8 is more likely to be associated with renal damage. In this study, we found that ANGPTL8 levels were higher in the MsPGN and FSGS groups. Patients diagnosed with MsPGN or FSGS are always refractory cases and are resistant to glucocorticoids. ANGPTL8 is a factor related to metabolism; therefore, nonimmune factors may play a substantial role in MsPGN and FSGS pathology.

Considering the findings of this investigation, further urine ANGPTL8 studies should address the mechanisms related to renal damage. Notably, as a small secretory glycoprotein, the source of ANGPTL8 detected in urine still needs to be identified. Whether ANGPTL8 can be synthesized and secreted in the kidney is a primary feature to investigate when studying the pathophysiological significance of ANGPTL8 in renal damage, and we are conducting related experiments. And, our study was lack of ANGPTL4 or ANGPTL3 assessment, which are very necessary to complete in our future working.

## Conclusions

Taken together, the findings of this study provide a preliminarily understanding of the serum and urine ANGPTL8 levels in patients with PNS and examined their association with the main disease indicators. This study aids in the understanding of PNS pathogenesis and is a basis for further exploration of potential therapeutic targets.

## Data Availability

The datasets generated during and/or analysed during the present study are available from the corresponding author upon reasonable request.

## Abbreviations

PNS: Primary nephrotic syndrome
HC: Healthy controls
24hUTP: 24 h urine total protein
ALB: Serumalbumin
CHOL: Cholesterol
TG: Triglycerides
LDL-C: Low density lipoprotein-cholesterol
BMI: Mass index
MCD: Minimal change disease
FSGS: Focal segmental glomerulosclerosis
MN: Membranous nephropathy
MsPGN: Mesangial proliferative glomerulonephritis
HDL-C: High density lipoprotein-cholesterol
CREA: Creatinine
Ur: Urea
UCr: Urinecreatinine
ELISA: Enzyme-linked immunosorbent assay
S.D.: Standard deviations
LDL: Low density lipoproteins
VLDL: Very low density lipoprotein
LPL: Lipid lipase
